# A case of cancer-associated retinopathy with chorioretinitis and optic neuritis associated with occult small cell lung cancer

**DOI:** 10.1186/s12886-019-1103-4

**Published:** 2019-05-02

**Authors:** William Carrera, Kathy A. Tsamis, Rajiv Shah

**Affiliations:** Wake Forest Baptist Medical Center, 1 Medical Center Boulevard, Winston-Salem, North Carolina 27157 USA

**Keywords:** Cancer-associated retinopathy, Small cell lung cancer, Chorioretinitis, Optic neuritis

## Abstract

**Background:**

Cancer-associated retinopathy (CAR) is associated with various malignancies, including small cell lung cancer (SCLC). It is difficult to recognize, but prompt diagnosis is crucial for the patient, as retinopathy may be a herald sign that precedes systemic manifestations by months, thus allowing early treatment of the underlying malignancy.

**Case presentation:**

We present a rare case of CAR with chorioretinitis and optic neuritis in a patient with occult SCLC. The patient presented with rapidly progressive peripheral field loss and photopsias with “prism-like” visual disturbances. Her symptoms stabilized with intravenous methylprednisolone, and her cancer was treated with carboplatin, etoposide and radiotherapy.

**Conclusions:**

This is the first reported case of SCLC-associated CAR to present with chorioretinitis. CAR can be a herald feature of SCLC, and early recognition of the disease should prompt a systemic evaluation for an occult malignancy, which may be critical for patient survival. Further understanding of CAR pathogenesis may offer potential avenues for treatment.

## Background

Small cell lung cancer results in roughly 29,000 diagnoses annually in the United States [1]. It is a highly aggressive tumor and prognosis for limited disease remains guarded, with cure rates of approximately 15–20%. The prognosis of extensive disease is dismal, and the overall SCLC survival rate is approximately 7% at 5 years [2, 3]. Prompt recognition of early clinical manifestations is crucial.

CAR is associated with numerous malignancies, including SCLC [4]. CAR was first reported by Sawyer in 1976, who identified three patients with rapidly progressive, binocular vision loss, retinal arteriolar attenuation, and degeneration of the photoreceptor and outer nuclear retinal layers [5]. Autoantibodies against recoverin were later identified in the sera of similar patients [6].

### Case presentation

A 49-year-old woman presented with 2 weeks of peripheral vision loss and intermittent, painless, ten-minute episodes of peripheral “prism-like” photopsias. Her vision loss progressed and became more persistent while intruding bitemporally towards central fixation. She denied other ocular or systemic symptoms. Her past medical history was notable for a 15-pack year smoking history. She had no personal or family history of ocular or autoimmune disease.

At her initial visit, her best-corrected visual acuity (BCVA) was 20/30 in the right eye (OD) and 20/25 in the left eye (OS) with intraocular pressures (IOP) of 11 and 12 mmHg, respectively. Anterior segment and fundus exam, as well as laboratory evaluation and neuroimaging, were unremarkable. Lumbar puncture demonstrated elevated protein with negative oligoclonal bands and normal IgG index. Her presentation was concerning for bilateral optic neuropathy, and she was treated with IV Methylprednisolone 1000 mg daily for 5 days. She noted that steroid treatment arrested progression of her visual symptoms.

**Fig. 1:**
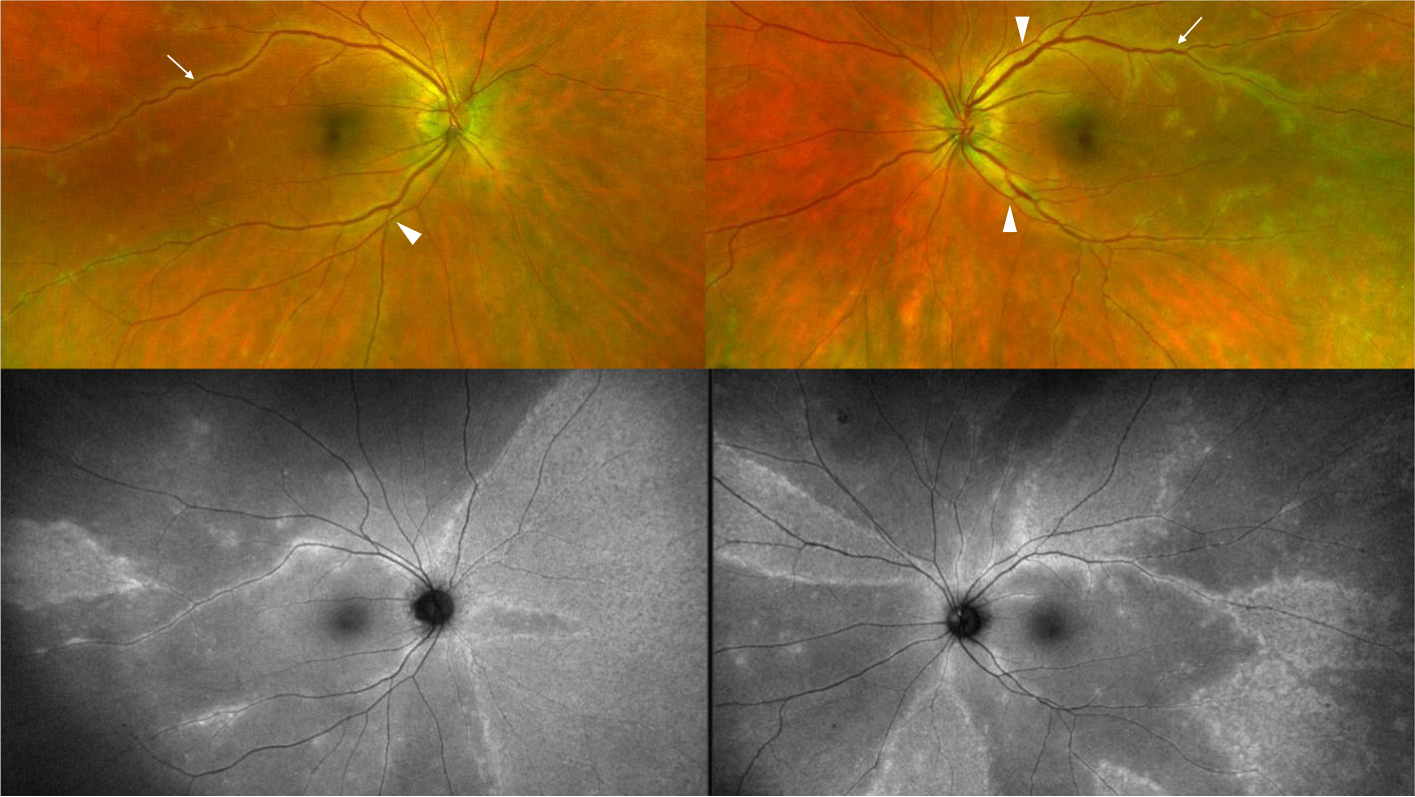
Optos color photographs OD and OS (above), demonstrating vascular attenuation (arrowheads) and sheathing (arrows). Optos fundus autofluorescence OD and OS (below) at 1 month after onset of symptoms

**Fig. 2:**
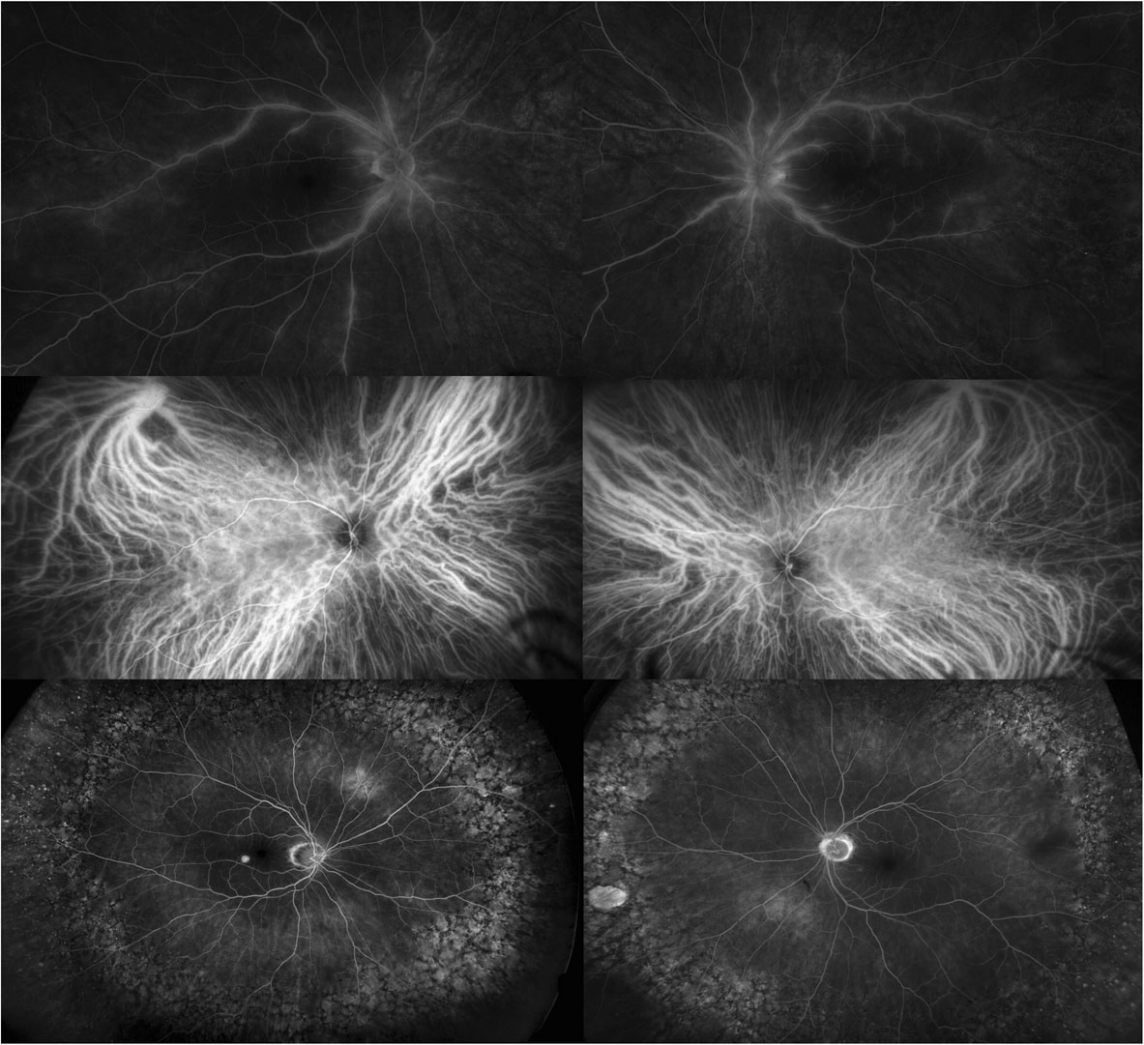
Fluorescein angiography (top) and ICG (middle) at 1 month after symptom onset, demonstrating hyperfluorescence consistent with leakage, and hypercyanescence with dilated choroidal vessels respectively. Fluorescein angiography at 3 min and 19 s OD and at 12 min and 3 s OS (bottom) at five-month follow-up demonstrating peripheral mottling and window defects

**Fig. 3:**
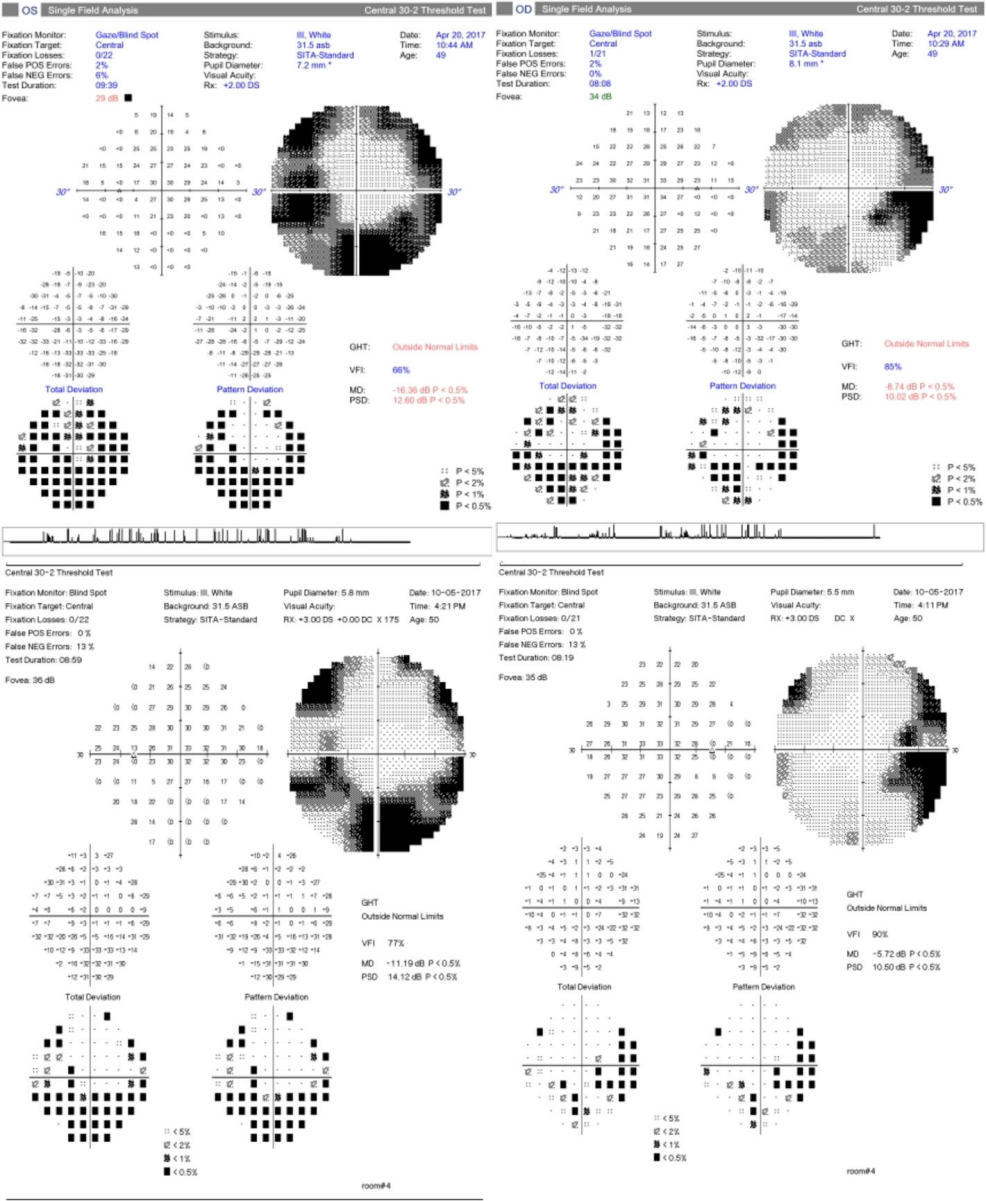
HVF 30–2 at 2 weeks after symptom onset (above) and at six-month follow-up (below), with a temporal defect OD and generalized peripheral constriction OS

At the one-month follow-up, she reported stability of her visual symptoms. BCVA was 20/30 in both eyes (OU) with IOP of 16 and 17 mmHg OD and OS, respectively. Exam was notable for trace vitreous cell OU, retinal venous sheathing and retinal whitening OU. Widefield Optos color fundus photos and autofluorescence (Fig. 1), exhibited peripheral regions of RPE hyperautofluorescence, demonstrating areas of photoreceptor and RPE degeneration. Fluorescein angiography (FA) demonstrated areas of perivascular hyperfluorescence that increased in intensity with time, consistent with leakage and retinal periphlebitis. Indocyanine green angiography (ICG) demonstrated choroidal hypercyanescence and dilated choroidal vasculature OU (Fig. 2). Humphrey visual fields (HVF) demonstrated a temporal field deficit OD and peripheral constriction with a central island remaining OS (Fig. 3), which corresponds to the retinal changes observed by fundus autofluorescence.

**Fig. 4:**
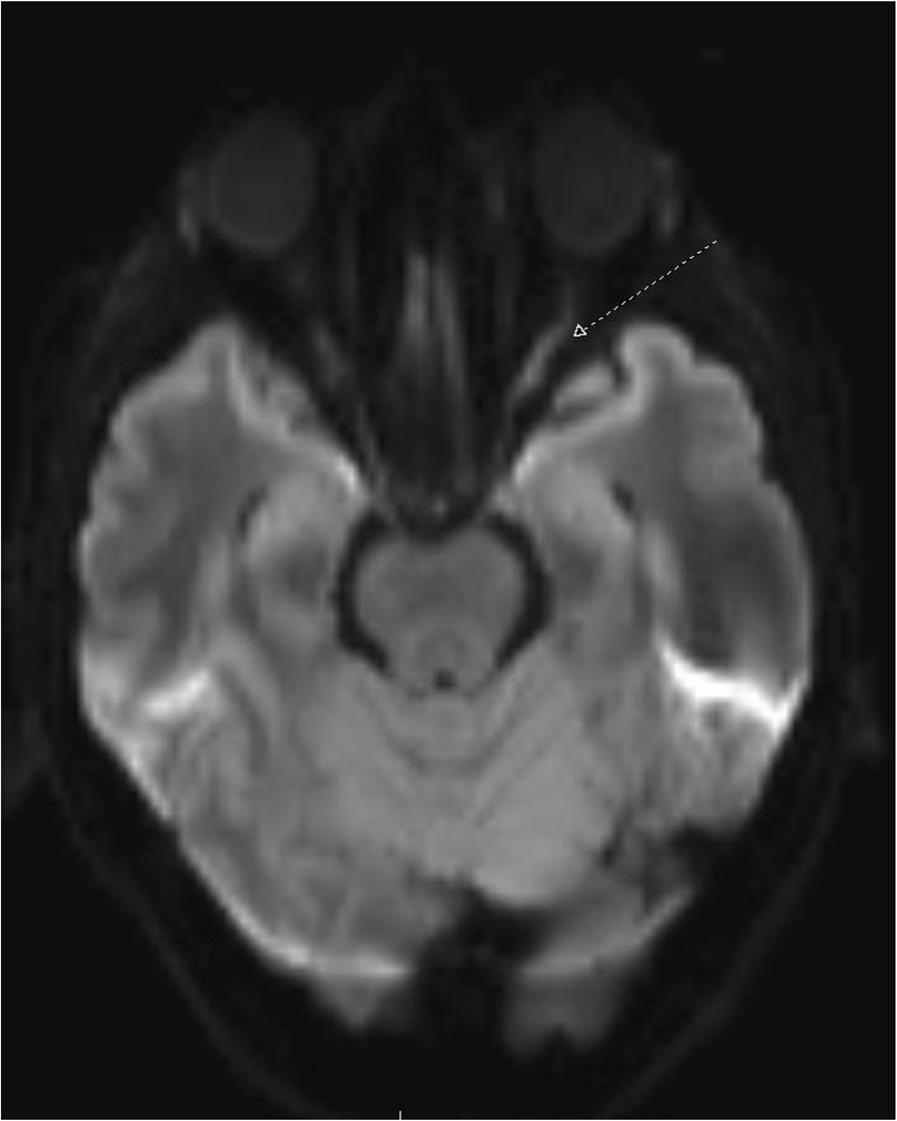
MRI of the brain demonstrating optic neuritis of the left optic nerve (dashed arrow)

She was admitted and treated with high dose IV methylprednisolone. Repeat MRI of the brain noted increased FLAIR signal, T2 enhancement and restricted diffusion of the left optic nerve, concerning for optic neuritis (Fig. 4). CT of the chest revealed mediastinal and left hilar adenopathy. Fine needle aspirate (FNA) biopsy revealed a poorly differentiated neuroendocrine carcinoma.

She was diagnosed with limited stage SCLC, chorioretinitis and optic neuritis consistent with CAR. An autoantibody panel was positive for anti-recoverin and negative for other CAR-associated antibodies. Electroretinography (ERG) was scheduled but was not obtained due to progression of her malignancy and worsening clinical course. She underwent treatment with carboplatin, etoposide and radiotherapy, but she declined prophylactic brain radiotherapy. She was maintained on Prednisone 60 mg daily for 12 weeks before being tapered by 10 mg weekly to 10 mg daily.

**Fig. 5:**
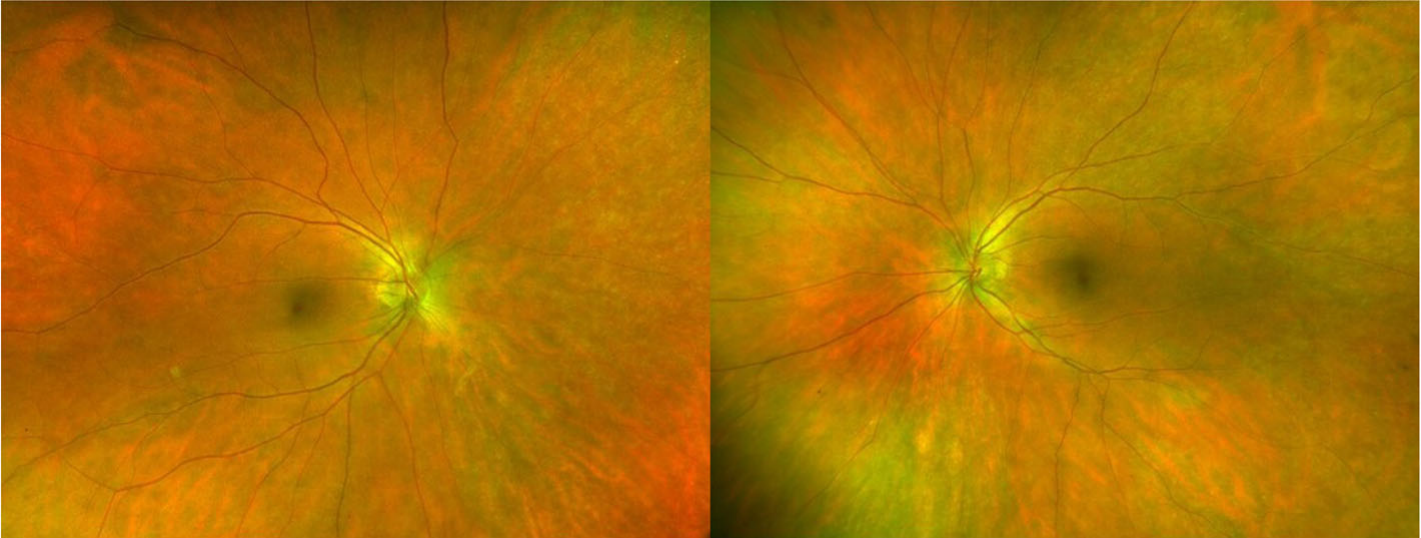
Optos color photographs OD and OS at six-month follow-up

At the six-month follow-up, her BCVA and HVF were stable (Figs. 3 and 5). Fundus exam demonstrated persistently attenuated vasculature, retinal whitening and development of choroidal and outer retinal scarring. She achieved and sustained remission until brain metastasis was detected 10 months after the initial onset of visual symptoms.

## Discussion and conclusions

CAR has a heterogeneous presentation. Common symptoms include generalized vision loss, blurry or dim vision, photopsia, nyctalopia and photophobia [7]. Symptoms are typically bilateral and rapidly progressive, and usually precede the diagnosis of SCLC by several months [7]. Exam findings commonly include retinal arterial attenuation, vitreous cell, retinal pigment mottling, and disc pallor. A small number of cases have demonstrated vasculitis or phlebitis [8–13]. ERG findings are nearly uniformly depressed or flattened [7]. Of numerous autoantibodies associated with CAR, anti-recoverin is among the most common, while prior cases with concomitant optic neuropathy have been known to manifest other autoantibodies, such as anti-CRMP-5 [12].

Recoverin is a 23-kDa calcium-binding protein that is expressed by photoreceptor cells, and that regulates rhodopsin light and dark adaptation [14, 15]. Recoverin is expressed by SCLC cells and is shed extracellularly during cell turnover and necrosis, thereby permitting autoantibody formation [15]. Within malignant cells, recoverin likely plays a role in calcium signaling and cell proliferation [16]. Recoverin is expressed by various benign and malignant tumors other than SCLC [17–19]. This suggests that additional processes are involved in anti-recoverin autoantibody formation beyond mere expression of recoverin by malignant cells. Inhibition of cytotoxic T lymphocyte antigen 4 (CTLA4) signaling is necessary for CAR pathogenesis, and it is likely that both humoral and cell-mediated components are involved [20].

Anti-recoverin autoantibodies enter retinal cells via endocytosis, and induce apoptosis, likely by inhibiting normal recoverin function, which leads to a rise in intracellular calcium that activates calcium-sensitive endonucleases and caspases [16, 21]. This mechanism is supported by work demonstrating that calcium channel blockade and dark adaptation rescues photoreceptors from apoptosis after administration of anti-recoverin antibodies [22]. Other antibodies that lead to intracellular stress can synergistically enhance this apoptotic process [16]. An apoptotic mechanism of retinal cell death is also consistent with the absence of retinal inflammation and the presence of autophagosomes and macrophages that has been observed on histologic preparations of retinas affected by CAR [5, 8, 23, 24].

Vascular compromise is a necessary component of CAR pathogenesis. In order for autoantibodies to access retinal targets, some breakdown of the blood brain barrier (BBB) must take place. The CSF of patients with CAR may contain elevated immunoglobulin, suggesting BBB permeability [25]. Histologically, retinas affected by CAR have shown perivascular lymphocytic invasion, typically without inflammation of the remainder of the retina [6, 9]. One case identified retinal venous capillary leakage, followed by surrounding areas of window defect on FA [9]. This may suggest that a vasculitic process allows initial retinal access to autoantibodies, and that subsequent RPE damage may exacerbate this process by allowing further access via choroidal vasculature. Murine studies have demonstrated that tumor-secreted vascular endothelial growth factor (VEGF) and placental growth factor (PlGF) act upon retinal VEFG receptor 1 to induce loss of pericytes within the retinal vasculature, thereby causing vascular leakage [26].

Our patient was noted to have periphlebitis on FA, dilated choroidal vessels on ICG, and demonstrated elevated CSF protein with negative oligoclonal bands and a normal IgG index, suggesting BBB breakdown and entry of serum immunoglobulin into the CSF. Other possible etiologies of her vision loss, such as a compressive lesion or hypercoagulability leading to ischemic optic neuropathy, were excluded based on imaging and laboratory evaluations, respectively. Most patients with SCLC-associated CAR do not exhibit overt vasculitis on fundoscopy or FA. Among the few reported cases of SCLC-associated CAR with retinal vasculitis, the case we present here is the first case to also demonstrate chorioretinitis [8–13]. This may support the hypothesized role of vascular compromise as a key step in CAR pathogenesis.

Further understanding of CAR pathogenesis may provide insight into this rare disease and reveal potential treatment modalities. The use of intravitreal anti- angiogenic agents, such as bevacizumab or ranibizumab, to inhibit the ability of tumor-secreted VEGF to compromise retinal vasculature and allow autoantibodies to access retinal targets offers an interesting, although untested, potential technique [26].

We present a case of SCLC-associated CAR with unique features including a combination of chorioretinitis and optic neuritis. This is the first case of SCLC-associated CAR with associated chorioretinitis. CAR is challenging to diagnose and treat. Data regarding disease epidemiology and long-term visual prognosis remains lacking. Further understanding of CAR pathogenesis may offer potential avenues for treatment. CAR can be a herald feature of SCLC, and early recognition of the disease should prompt a systemic evaluation for an occult malignancy, which may be critical for patient survival.

## Abbreviations

BBB: Blood-brain barrier
BCVA: Best-corrected visual acuity
CAR: Cancer associated retinopathy
CTLA-4: Cytotoxic T-lymphocyte-associated protein 4
ERG: Electroretinography
FA: Fluorescein angiography
FNA: Fine needle aspirate
HVF: Humphrey visual field
ICG: Indocyanine green angiography
OD: Oculus dexter, right eye
OS: Oculus sinister, left eye
OU: Oculus uterque, both eyes
PlGF: Placental growth factor
SCLC: Small cell lung cancer
VEGF: Vascular endothelial growth factor
